# Case Department

**Published:** 1888-07

**Authors:** 


### CASE DEPARTMENT.

I.—An Obscure Case of Chronic Glanders.—Gray mare, 9 years, 15.2 hands, rather well bred, but shows a lymphatism which does not correspond with the general formation of the animal.

April 28th. Mare is somewhat dejected, slightly emaciated, skin dry and hairs rough. Temp. 100½° F., P. 36, Resp. 18—the latter prolonged on expiration. Has a very slight, somewhat viscous, discharge from both nostrils; submaxillary lymphatics hard, rather low in position, and somewhat masked by a peripheral œdema. On inside of near thigh a small yellowish red scab, which, on removal, leaves a small, unhealthy ulcer. The latter covers at once with a similar scab, and for several weeks shows no tendency to heal. On same thigh, a small nodule the size of a large bullet, which, on puncture with a bistoury, gives a few drops of a viscous pus, and then follows the course of the first ulcer.

*Diagnosis.*—Chronic Glanders.

The mare was placed on low diet and purged with nine drachms of aloes. In four days the purge was renewed, acting as the first did, freely. The discharge becomes intermittent, slight on some days and entirely absent on others. The submaxillary glands lose the œdematous surrounding, appear hard but not nodulated and rather low down. The ulcers on thigh remain the same.

Matter from the nostrils was inoculated on the sides of the lip and on the nose of the same mare, and for a few days produced a yellowish red oily discharge, followed by cicatrization, but the yellowish scabs remained for some time. Inoculation of the same discharge into two guinea-pigs produced death in one at the end of five days, the autopsy showing a number of tubercles in the viscera. The second pig died from other causes. On the fourteenth day some increase in discharge, the mucous membranes of nostrils varnished and leaden, and by the aid of an opthalmascope three small tubercles can be seen high on right side of the septum nasi. These, with the increased discharge, disappear on the second day. The mare was again purged with aloes, but the symptoms remain as at first. One week later the manger, nostrils, breast and forelegs were found covered with a considerable quantity of clotted blood. No further symptoms were developed. Faint sibilant rales could at times be heard on ausculting the lungs. There was never any development of febrile symptoms.

May 26th. Mare was pitted and opened. Both lungs were farcied with small, hard, indurated tubercles, in some of which deposits of chalk salts had taken place. On section of the head a moderate collection of a viscous bright colored pus was found in the right hand frontal and superior maxillary sinuses, from which the epithelium was denuded; the septum and nasal fossæ in the upper half was a mass of old indurated cicatrices, unhealthy small ragged ulcers with very little discharge, and tubercles.

R. S. Huidekoper, *Veterinarian.*

II.—A Peculiar Case of Rheumatism.—In November last I was called to attend a horse and received the following history: "Horse was attacked about three weeks ago, and for some time treated by an unqualified practitioner without benefit. I then sent for ———, who pronounced his trouble to be situated back of the ears, and blistered him to bring the "matter" to the surface. The horse is getting worse daily, eats but little, is very much afraid of the shadows cast by a lantern and grunts if you point your finger at him."

I found the pulse but little accelerated, of good volume and steady, temperature up about a degree, the cervical muscles hard and very painful, a like condition obtaining with the posterior portion of the great dorsal and the fascia lata muscles. The sheath was swollen and an œdematous swelling extended thence on the inferior abdominal region to the xiphoid appendage of the sternum. There was a little tenderness on pressure over the intercostals, but no fixation of them or abdominal respiration pointing to pleurisy. The animal was almost unable to move in the stall, and could not get his head down to his manger. A careful rectal examination gave negative results.

*Diagnosis.*—Muscular rheumatism. Treatment: Ac. Salicyl-Colchici pulv. with pot. nitrates and opium, the horse to be rubbed from head to foot with soap liniment containing oil of wintergreen and to be thoroughly groomed daily. Result most gratifying. The tenderness rapidly disappeared, the swelling of the sheath and belly was removed, the appetite returned, and the animal laid down and rested comfortably, and with this amelioration the nervous symptoms disappeared; gentle exercise was given, and everything was pointing to a speedy recovery, when all the symptoms returned, aggravated in intensity and accompanied by croupous discharges per rectum (I think this due to the administration of the saltpetre, as I have seen it in several cases where that remedy was pushed). Again the symptoms became somewhat ameliorated, but with no abatement of the nervousness and with the addition of intense pain and swelling of the flank muscles. Treatment continued with addition of hot fomentations to flanks and some bromides. At length the horse got down and was unable to rise. I let him lie about thirty-six hours, then put slings under him, and he made so little effort to stand in them that the owner brought me an axe and requested me to destroy him. After renewed efforts we got him to stand and found that he had "barked" himself pretty severely about the flanks and anterior crural region; indeed, the skin sloughed; *with the sloughing the symptoms disappeared*, and the animal is now well, with no trace of nervousness. The next case I have of this kind I shall either fire in points over a considerable area or insert setons to form avenues of discharge.

Woodbury, N. J. T. B. Rogers, D. V. S.

III.—A Castor-oil fed Lipoma.—Concerning the growth and development of this specie of tumor, the following case, which came under my notice, may be worthy of description:

A gentleman first noticed last September, while bridling his five-year old bay mare, that there was a small tumor, the size of a bean, on the anterior border and at the base of the conchal cartillage. It was movable under the skin and did not seem to increase in size, but the owner, wishing its removal, took the advice of a neighbor and applied castor oil daily for about three weeks. In the meantime it began to grow rapidly, and at the time he stopped using the oil it was about the size of a hen's egg. After the use of the oil was discontinued the tumor stopped growing and remained the same until the middle of January, when by request of owner I extirpated it. It was loosely attached to the skin, but quite firmly attached at its base to the fibrous tissue covering the cartilage. The wound was dressed antiseptically and soon healed. On examination of tumor I found it covered with a thin fibrous capsule, and the tissue within was so saturated with oil that it oozed out freely on pressure. Now the question arises, Did the oil act as pabulum for this growth, or did the manipulation during treatment stimulate its growth? The former would seem the more rational theory, because, after the oil treatment, the surface was continually abraded until extirpation, without any perceptible increase in size of the tumor.

A Large Abscess the Result of a Punctured Wound of the Abdomen.—The proprietor of a stock farm came for me in great haste, saying that a brood mare had received a wound in the abdomen from a pitchfork, both tines entering the left flank. She ran six or eight rods with the fork still clinging to her. When I arrived, the mare was breathing hurriedly, had a quick pulse and a general muscular tremor indicative of much pain. I immediately administered a ball containing opii pulvus oz. ii, followed by 30 minim doses of Tr. aconite every hour. There was little or no hemorrhage from the external wounds, and she soon becoming quiet, I left her with orders to keep her on sloppy food. The next day two small circumscribed swellings were to be seen at the points of puncture. Sho had anorexia, tenderness of the abdomen. Temperature 103½, pulse 80, respiration 20; evidently suffering from peritonitis. Gave opiates and fed on gruel of bran and oatmeal. The symptoms gradually subsided and temperature became normal. Within five days swellings all disappeared and she was put on her regular diet. Three weeks after, owner wished me to call again and see the mare; on seeing her, found a very hard swelling near one of the punctures; this was first noticed by owner about a week before, when the mare began to have loss of appetite, grew poor, and evinced pain, especially when moving, near hind limb; temperature 103. I diagnosed an abscess deeply seated under the muscles. To be sure, I thrust an exploring needle into the swelling, and about a teaspoonful of thick pus and some very dark blood oozed out. I then clipped off hair over the swelling and applied a thick mustard paste. On the fourth day the abscess began to "point" through the track made by the needle; I could pass a probe through this track to a depth of five inches. Then with a bistoury, carefully guarded with a director, I made an opening through the muscles into the abscess and got about a pint of thick laudable pus. On exploration with the finger, I found an irregular cavity, nearly round, and having an average diameter of about six inches. The confined pus had evidently dissected a part of the aponeurosis of the transverse abdominal muscle from the muscles external to it, thus forming the pus cavity. The next day after operation the temperature was normal, appetite improving, and the lameness and soreness gradually disappeared. Wound was dressed twice daily with three per cent. phenic acid solution; it discharged profusely and cavity is now fast disappearing.

In this case I am very confident that abscess was not caused by any foreign body introduced at the time of the wound, for the fork tines were bright and clean and entirely free from rust. Evidently, a slight hemorrhage and an unabsorbed blood clot furnished the nucleus for this abscess, and undoubtedly the simple track made by the exploring needle prevented results that might have proven serious had the abscess perforated its thin inner wall and had broken into the abdominal cavity.

MONTPELIER, Vt. L. C. WAKEFIELD, D. V. S.

